# Approaching Polymer Dynamics Combining Artificial Neural Networks and Elastically Collective Nonlinear Langevin Equation

**DOI:** 10.3390/polym14081573

**Published:** 2022-04-12

**Authors:** Luis A. Miccio, Claudia Borredon, Ulises Casado, Anh D. Phan, Gustavo A. Schwartz

**Affiliations:** 1 Centro de Física de Materiales (CSIC-UPV/EHU)—Materials Physics Center (MPC), P. M. de Lardizabal 5, 20018 San Sebastian, Spain; borredon.claudia@gmail.com; 2 Donostia International Physics Center, P. M. de Lardizábal 4, 20018 San Sebastian, Spain; 3 Institute of Materials Science and Technology (INTEMA), National Research Council (CONICET), Colon 10850, Mar del Plata 7600, Argentina; ulisescasado@fi.mdp.edu.ar; 4 Faculty of Materials Science and Engineering, Phenikaa University, Hanoi 12116, Vietnam; anh.phanduc@phenikaa-uni.edu.vn; 5 Phenikaa Institute for Advanced Study (PIAS), Phenikaa University, Hanoi 12116, Vietnam

**Keywords:** QSPR, dynamics prediction, polymers, artificial neural networks, smart design

## Abstract

The analysis of structural relaxation dynamics of polymers gives an insight into their mechanical properties, whose characterization is used to qualify a given material for its practical scope. The dynamics are usually expressed in terms of the temperature dependence of the relaxation time, which is only available through time-consuming experimental processes following polymer synthesis. However, it would be advantageous to estimate their dynamics before synthesizing them when designing new materials. In this work, we propose a combined approach of artificial neural networks and the elastically collective nonlinear Langevin equation (ECNLE) to estimate the temperature dependence of the main structural relaxation time of polymers based only on the knowledge of the chemical structure of the corresponding monomer.

## 1. Introduction

The mechanical behavior of polymeric materials is key to several industries such as aerospace, transport, energy, and construction, among many others [1,2,3,4,5,6,7]. Since the mechanical properties, together with the overall service life performance of these materials, are directly related to their dynamics, the knowledge of the latter becomes highly relevant. For instance, in transport and aerospace industries, some materials are expected to be able to perform well through wide ranges in terms of frequency, presenting a low rolling resistance and at the same time a large dissipation of energy during a braking period (processes that correspond to approximately 10^−2^ Hz and 10^4^–10^7^ Hz, respectively) [8,9,10,11]. Therefore, for obtaining the required on-service behavior, adequate polymer selection is combined with the fine-tuning of several other properties such as processability, durability, and energetic efficiency. Molecular dynamics determines such mechanical properties of the compound, and it is usually described in terms of a characteristic relaxation time and its temperature dependence. The experimental window of these relaxations (that can extend over several decades) imposes the necessity of a combination of techniques (such as broadband dielectric spectroscopy (BDS), dynamic light scattering (DLS), or dynamic mechanical analysis (DMA)), in turn converting this practice into a costly and time-consuming process that could increase development costs.

Nevertheless, some theoretical approaches can help when designing and developing new materials since there is no prior information about their dynamics before synthesizing and characterizing them. Among these approaches, the elastically collective nonlinear Langevin equation (ECNLE) [12,13,14] theory was developed and successfully applied to describe the molecular dynamics of various amorphous materials. This model solely relies on the knowledge of the glass transition temperature (*T_g_*), which requires a non-negligible amount of time and resources to be determined when unknown. However, recent advances in the field of artificial neural networks (ANN) [15,16,17] enable the estimation of the glass transition temperature of polymers based only on the monomer’s chemical structure.

In this work, we combine theoretical and numerical approaches to estimate, from a representation of the chemical structure of amorphous acrylates, their glass transition temperature and the temperature dependence of the structural relaxation time. Firstly, we codify the chemical structure of the compounds using the Simplified Molecular Input Line Entry System (SMILES) [18,19] representation and employ it as an input for a neural network algorithm that would output an estimation of the polymer’s *T_g_*; then, we exploit this information as an input for the ECNLE to theoretically compute the trajectory of the molecular dynamics of the structural relaxation process, expressed as the temperature dependence of its relaxation time. We propose this approach as a tool to speed up research and development in the field of polymeric materials.

## 2. Methods and Theoretical Background

In this section, we explain the characteristics of the dataset, the process that the data undergo, the ANN’s architecture, and how it is tuned. In addition, we include a description of how ECNLE theory is applied to the estimation of the acrylates’ dynamics.

### 2.1. Dataset

We employed a cured dataset composed of about 200 atactic polyacrylates and their corresponding *T_g_* values above chain length saturation [20,21,22,23] (see Table S1). These acrylates’ monomer units were codified using a Simplified Molecular Input Line Entry System (SMILES) [18,19] and converted into binary matrices, which are then fed to the ANN.

The external control set was composed of those polymers for which the experimental dynamics was published. These data are essential since we want to compare the predicted dynamics against the experimental dynamics. Table SI2 shows the parameters of the Vogel–Fulcher–Tammann (VFT) equation that fits the corresponding observed dynamics together with the references the data were taken from.

### 2.2. Chemical Structure Encoding

As we proposed in recent works [15,16,17], to consider the structure and composition of the monomeric units, we transformed the chemical structures into linear strings using SMILES [18,19]. Then, we converted these strings into binary matrices using a one hot encoding algorithm [24] and a dictionary (composed by the following list of symbols: ‘(’, ‘O’, ‘C’, ‘=‘, ‘c’, ‘S’, ‘F’, ‘N’, ‘X’, ‘2’, ‘d’, ‘1’, ‘#’, ‘]’, ‘/’, ‘)’). Section S3 in Supplementary Materials provides a brief explanation of this encoding process.

### 2.3. ANN’s Architecture and Optimization

**Figure 1:**
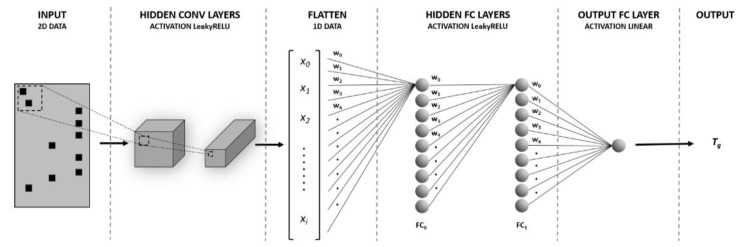
Schematic picture of the artificial neural network employed for predicting the glass transition temperatures of acrylates.

We used convolutional neural networks fed with the polyacrylates’ monomeric structures (codified into binary matrices) and the corresponding glass transition temperatures. Figure 1 shows a schematic view of the ANN’s architecture: the monomer structure is codified (through a one-hot encoding process applied on its SMILES string) into a 2D matrix which is then fed to convolutional layers to extract relevant chemo-structural features; the result is flattened into a 1D vector ($X\in R^{n}$) feeding two fully connected layers (FC_0_ and FC_1_) with LeakyReLU activations. Section S4 in Supplementary Materials provides more details about the neural network architecture. We compared several combinations of hyperparameters to achieve the best possible performance for the ANNs. Such comparison among ANNs was based on the raw performance (minimum relative error) obtained on the dataset. A dropout [25] algorithm was used, with dropping probabilities ranging from 0 to 0.3. Finally, the last hidden layer (FC_1_) was connected to a single neuron with a linear activation function responsible for providing the glass transition temperature value.

As done in previous works [15,16,17], we implement the mean absolute relative error as a loss function in the training process to ensure equal weighting of low and high *T_g_* data values. Given *E_i_* (experimental *T_g_*), *F_i_* (forecasted *T_g_*), and the number of acrylates in one mini-batch *m_x_*, we define the mean absolute percentage error as

(1) $$Loss=\frac{100}{m_{x}}\cdot \overset{m_{x}}{\underset{i=1}{\sum }}\left|\frac{E_{i}-F_{i}}{E_{i}}\right|$$

We adopt a mini-batch gradient descent technique to minimize the loss function, using an Adam optimizer [26] with a learning rate (lr) of 0.0001 for speeding up the convergence and mini-batches of 20 acrylates each.

As usual, the data were randomly divided into test and train subsets during the training process, and no enforcement of any preference in the way the data are split was applied. In addition, an external control group (independent from the previous subsets) was formed for studying polymer dynamics through ECNLE theory. ANN details are summarized in Table 1 and Figure 1 (more details are provided in Section S4 of the SI).

### 2.4. Nonlinear Langevin Equation

ECNLE theory describes glass-forming liquids using a hard-sphere fluid [12,13,14] of volume fraction $\Phi =\rho \pi d^{3}/6$, where *d* is the particle size and ρ is the number of particles per volume. The local dynamics takes account of a tagged particle considering: (1) interactions with its nearest neighbors, and (2) cooperative motions of particles beyond the first shell. The dynamics is quantified by the dynamic free energy [12,13,14], $F_{dyn}\left(r\right)=F_{ideal}\left(r\right)+F_{caging}\left(r\right)$, where r is the displacement, $F_{ideal}\left(r\right)$ represents the ideal fluid dynamics and $F_{caging}\left(r\right)$ characterizes the local state of a particle subject to caging forces conditioned by the structural features of the system. When the fluid has a sufficiently large density (Φ≥0.432) or is in a low enough temperature, the motion of particles is restricted within a particle cage of radius $r_{cage}$ and a barrier in $F_{dyn}\left(r\right)$ emerges with a barrier height given by $F_{B}=F_{dyn}\left(r_{B}\right)-F_{dyn}\left(r_{L}\right)$, where $r_{L}$ is the localization length of the particle and $r_{B}\;$ is the barrier position. The escaping of a particle from its cage produces a collective elastic long-range rearrangement of the molecules in the fluid, whose energy contribution is given by a sum over harmonic oscillators which is described in Section S5 of the SI. Once the local and elastic dynamics are defined and the harmonic curvatures at $r_{B}$ and $r_{L}$ (respectively $K_{0}$ and $K_{B}$, see SI) is estimated, we calculate the structural relaxation time using Kramer’s theory

(2) $$\frac{\tau }{\tau_{s}}=1+\frac{2\pi }{\sqrt{K_{0}K_{B}}}\frac{k_{B}T}{d^{2}}exp\left(\frac{F_{B}+F_{e}}{k_{B}T}\right)$$

where $\tau_{s}$ is a short time scale [12,13,14]. As the above calculations provide τ(Φ), we use a simple thermal mapping $T=T_{g}+\left(\Phi_{g}-\Phi \right)/\beta \Phi_{0}$, where *T_g_* is the dynamic glass transition temperature defined by $\tau \left(T_{g}\right)=100\;s$, $\Phi_{g}$ is the volume fraction when $\tau \left(\Phi_{g}\approx 0.6157\right)=100\;s$, $\Phi_{0}\approx 0.5\;$ is a characteristic volume fraction, and $\beta \approx 12\times 10^{-4}{\;K}^{-1}\;$ is an effective thermal expansion coefficient considered constant for all amorphous materials. Further details to derive the theory are given in the Supplementary Materials and elsewhere [12,13,14].

## 3. Discussion

**Figure 2:**
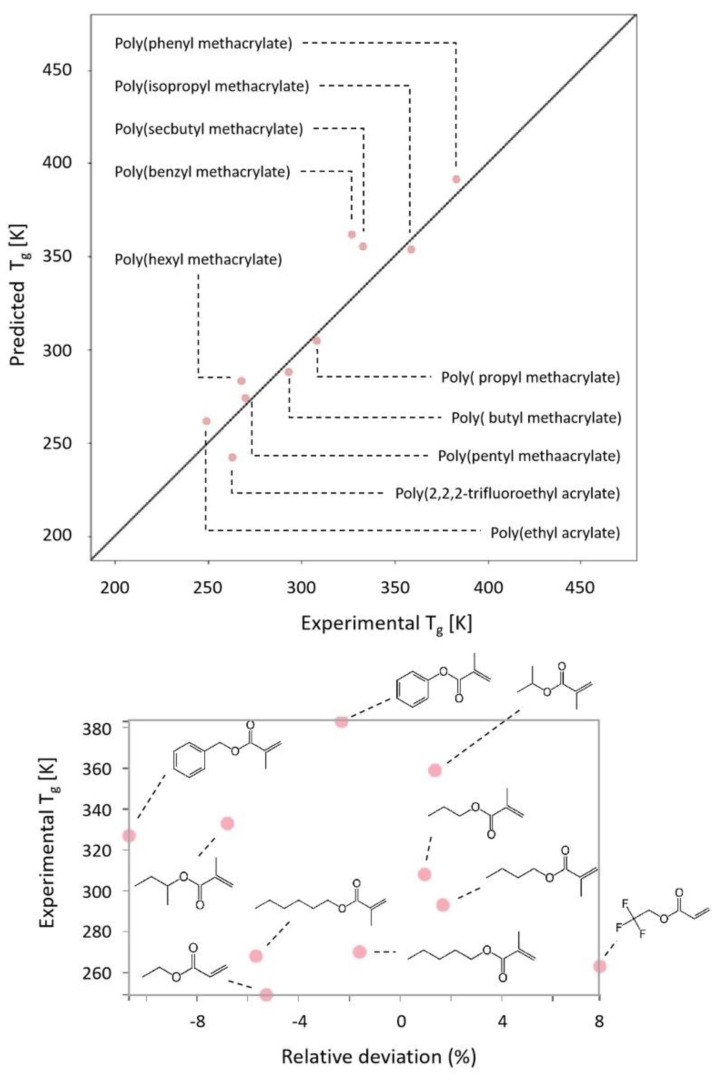
Predicted vs. experimental glass transition values obtained from the trained ANN on the external control group of acrylates. Relative deviations are shown below with the corresponding monomeric chemical structures.

Figure 2 shows predicted vs. experimental values of the glass transition temperature for the external control set of polyacrylates, as obtained with our trained ANN (see also Figure S2 for the training and internal test sets). We obtained mean absolute percentage errors of 4.3% (training set), 8.5% (validation set), and 4.5% (control set). In comparison with other neural network approaches that we have used in the past [15], the relative number of parameters (and, therefore, calculations) is reduced thanks to a convolutional approach (due to the stride convolution operation that tosses out parts of the input image). It is worth remembering here that we are feeding the ANN only with the monomer chemical structure without any other physical or chemical input data (neither measured nor calculated).

As shown, the ANN does capture the relationship between the chemical structure and the glass transition temperature of the polyacrylates all along the 200–400 K range (see also Figure S2). The individual relative deviations in the external control group are within (or close to) a 10% margin (see Table S1), in agreement with the observed values for the internal test. More details on the obtained relative deviation for the different chemical structures are depicted in Figure S3. Aside from the obtained low errors, our aim is not only to predict the *T_g_*, but also to obtain some insight into the dynamics of the polymers under study. For this purpose, the predicted glass transition temperatures are used as input for ECNLE theory, thus creating a hybrid ANN-theory approach for yielding a possible relaxation area (in terms of log ($\tau {)}_{\;}\;\mathrm{vs}.\;1000/T$).

**Figure 3:**
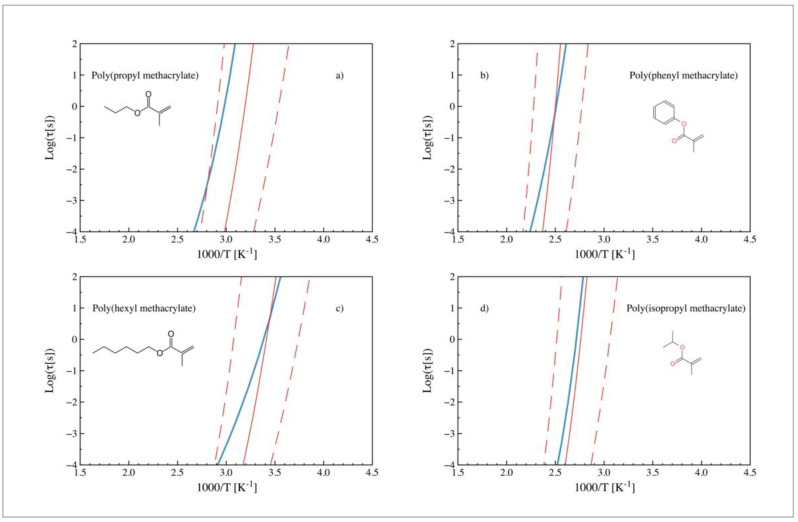
Experimental (blue) and predicted (red) relaxation times (obtained from ECNLE theory) vs. 1000/T. Dashed lines stand for the confidence interval corresponding to the typical deviation in the ANN prediction (10% relative error): (**a**) Poly (propyl methacrylate) [28], (**b**) poly (phenyl methacrylate) [30], (**c**) poly (hexyl methacrylate) [27], and (**d**) poly (isopropyl methacrylate) [30].

Hence, Figure 3 shows the temperature dependence of the alpha relaxation times for (a) Poly (propyl methacrylate), (b) Poly (phenyl methacrylate), (c) Poly (butyl methacrylate), and (d) Poly (isopropyl methacrylate). Blue lines represent the experimental values, reported elsewhere [27,28,29,30,31], while dashed lines represent the range of relaxation times obtained by ECNLE theory (from ANN’s predicted *T_g_* values), including error bands for *T_g_* ± 10% (corresponding to the maximum relative error on the external control set). As shown, the predicted relaxation region is very close to the experimental observations, having, therefore, an acceptable agreement (especially considering that only the chemical structure of the monomer is used as input).

In some cases, as for poly (phenyl methacrylate) or poly (isopropyl methacrylate) (see Figure 3b,d), the glass transition temperature is well predicted, but the curvature of the estimated dynamics deviates from the experimental values. In some other cases, as in Figure 3a,c, the deviations are even more pronounced. Therefore, despite being inside the proposed confidence interval, the curvature obtained from ECNLE theory does not follow the experimental dynamics. This behavior is most likely related to the assumption that local and collective dynamics correlate to each other for all materials in the same way (which is an excellent approach in terms of not needing any other inputs to obtain an approximated relaxation map but tends to oversimplify the behavior of the materials). In particular, local and collective dynamics in Equation (2) are summed with equal weights (i.e., the ratio of prefactor equal to 1). It has been shown [32,33] that ECNLE calculations gain accuracy by weighting the collective elastic contribution with a parameter a≠1, to change its relative importance in the glass transition process. The new adjusted elastic barrier is $F_{e}\to a^{2}F_{e}$ and it modifies the structural relaxation time in Equation (2) as

(3) $$\frac{\tau_{\;}}{\tau_{s}}=1+\frac{2\pi }{\sqrt{K_{0}K_{B}}}\frac{k_{B}T}{d^{2}}exp\left(\frac{F_{B}+a^{2}F_{e}}{k_{B}T}\right)$$

The parameter *a* strongly influences the structural features of the model (value of $\Phi_{g}$ and the thermal mapping), as it accounts for the non-universal effects on the collective motions of molecules due to conformational and chemical complexities. It was empirically observed that the *T_g_* is typically inversely proportional to the scaling parameter *a* [13]. Figure S4 shows the glass transition temperature dependence of the model adjustable parameter *a* for several polymers and glass formers. Although the correlation is not strong, there is a clear trend indicating an increment of the parameter *a* upon decreasing glass transition temperature. Thus, we can estimate the scaling parameter *a* based on the *T_g_*.

**Figure 4:**
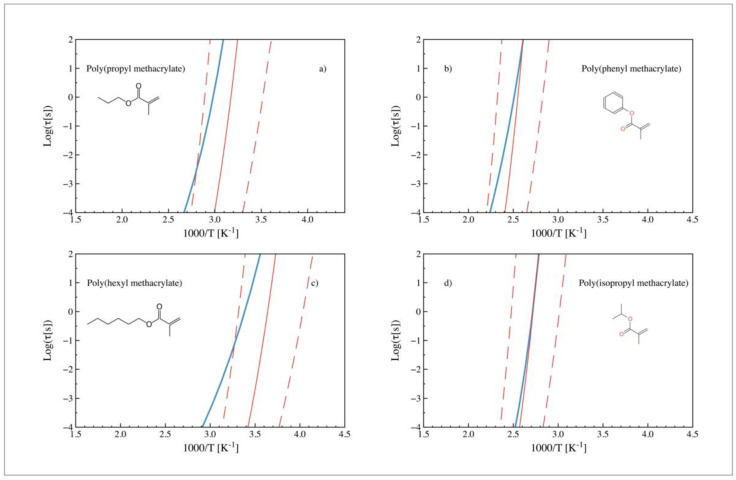
Experimental (blue) and predicted (red) relaxation times (obtained from ECNLE theory) vs. 1000/T after introducing the scaling parameter (*a*). Dashed lines stand for the confidence interval corresponding to the typical deviation in the ANN prediction (10% relative error). (**a**) Poly (propyl methacrylate), (**b**) poly (phenyl methacrylate), (**c**) poly (hexyl methacrylate) and (**d**) poly (isopropyl methacrylate).

**Figure 5:**
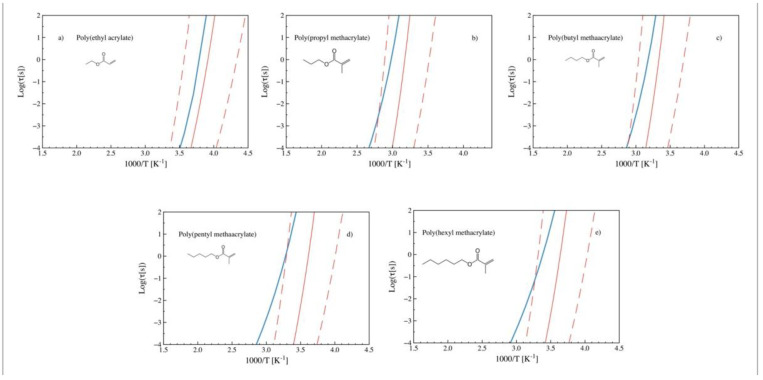
Experimental (blue) and predicted (red) relaxation times (obtained from ECNLE theory) vs. 1000/T (n-alkyl acrylates, with n ranging from 2 to 6). The corresponding monomeric chemical structures are also shown. (**a**) Poly (ethyl acrylate) [27], (**b**) poly (propyl methacrylate), (**c**) poly (butyl methacrylate), (**d**) poly (pentyl methacrylate) [27], and (**e**) poly (hexyl methacrylate) [27]. The plots correspond to predictions after introducing the scaling parameter (*a*) for linear tailed polymers.

Figure 4 shows the temperature dependence of alpha relaxation times for the same polymers as Figure 3 after introducing the scaling parameter (*a*). The predicted relaxation times change their curvature, displaying a better agreement (for cases b and d) with the experimental observations. In the case of poly (propyl methacrylate), no further improvement is perceived. It is also observed that, in the case of polymers with linear alkane tails, the experimental-predicted agreement appears to decrease as the length of the tail increases. As shown in Figure 5 (b) poly (propyl methacrylate) and (c) poly (butyl methacrylate) already reflect this trend, which intensifies for (d) poly (pentyl methacrylate) and (e) poly (hexyl methacrylate), while it is much smaller for (a) poly (ethyl acrylate).

Fragilities and dynamics data of members of the polyacrylates family have been obtained from mechanical and dielectric data by several authors [33,34,35,36,37,38,39,40,41,42]. From this experimental point of view, the increase in the length of the alkyl chain causes a strengthening effect. The variation of fragility (*m*) with the length of the alkyl chain appears to have three ranges: for less than three atoms, *m* is nearly constant; between three and five atoms, it drastically decreases; and, for more than five atoms, *m* slowly decreases. Moreover, Balabin studied the enthalpy difference between conformations of normal alkanes and showed that n-alkyl chains are more and more flexible as the chain length increases [43]. In addition, some local order structure gradually develops as the carbon number in the side chain increases due to a self-assembly process that forms supramolecular systems such as “hairy rods” [44,45].

Finally, it has also been reported that nanophase separation of incompatible main and side-chain parts occurs in amorphous side-chain polymers with long alkyl groups (for polymers with 4 or more C atoms in the side chain) [46,47,48,49]. Considering that the cooperative dynamics changes if the confinement size becomes comparable to the size of cooperatively rearranging regions (CRRs), these crystalline regions could affect the relaxation, thus creating a hindered glass transition [48]. Published results indicate that the CRR size for alkyl sequences is in the range of one nanometer [50,51,52].

A more detailed view of this effect on the prediction differences with the experimental data can be observed in Figure 5, where the relaxation maps of a series of alkyl-acrylates are presented. As shown, the predictions progressively deviate from the experimental curves as the side-chain length increases. Deviations in polymers with two or three atoms in the tail are almost exclusively related to deviations in the *T_g_* predicted by the ANN, while for longer chains, a difference in the predicted curvature is additionally noticed. It can be argued that the proposed approach yields acceptable predictions up to four or five atoms in the linear chain.

These predicted and experimental results can be reconciled by considering the ECNLE theory assumptions, which predicts the material dynamics in terms of a fluid composed of hard spheres and does not consider other processes (such as packing density, induced crystallization or nanophase separation). Therefore some deviations are expected from the experimental observations in these polymers where other processes occur. These deviations are related to the typical relaxation length of the alpha relaxation, which is in the nanometer range for these materials.

**Figure 6:**
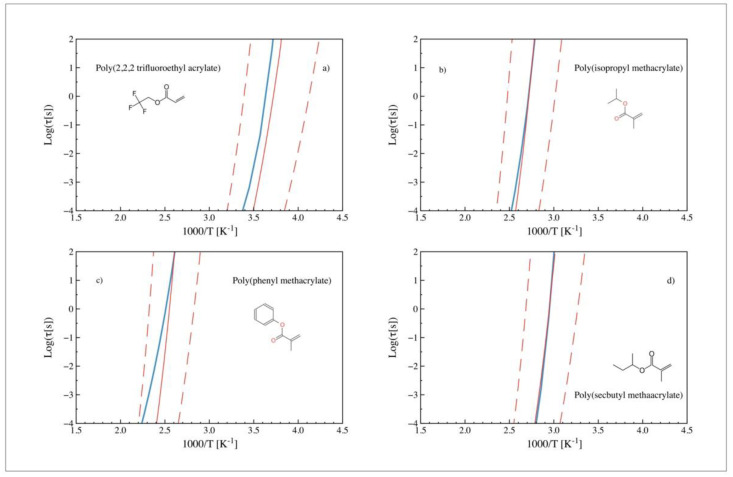
Experimental (blue) and predicted (red) relaxation times (obtained from ECNLE theory) vs. 1000/T after introducing the scaling parameter (*a*) for nonlinear tailed polymers. The corresponding monomeric chemical structure are also shown. (**a**) Poly (2, 2, 2 trifluoroethyl acrylate) [31], (**b**) poly (isopropyl methacrylate) [29], (**c**) poly (phenyl methacrylate), and (**d**) poly (secbutyl methacrylate) [30].

We can move further by analyzing the experimental-predicted dynamics relationship for polymers where the side-chain length effects are not present. In that sense, Figure 6 shows experimental (blue) and predicted (red) relaxation times obtained from ECNLE after introducing the scaling parameter *a* for nonlinear tailed polymers. Poly (2, 2, 2 trifluoroethyl acrylate) (a), poly (isopropyl methacrylate) (b), poly (phenyl methacrylate) (c), and poly (secbutyl methacrylate) (d) present a much better agreement than the long linear tailed polymers (such as pentyl or hexyl methacrylates).

For this joint theoretical/numerical approach, we have two sources of uncertainty: on the one hand, the prediction of the *T_g_* by the ANN; on the other hand, the accuracy of the ECNLE model to follow the temperature dependence of the relaxation times (i.e., fragility). Although the errors in both cases are not significant, there is still some room for improvement. The accuracy of the ANN can be improved by increasing the size of the training set; especially if we include polymers with chemical features similar to those we want to predict. In the case of the ECNLE model, a better understanding of the dependence of the parameter ‘*a*’ with the chemical structure or the glass transition temperature would improve the predicted fragility.

In summary, and from a chemical structure point of view, many different factors have been reported to affect the glass transition and the polymer dynamics, thus increasing the difficulties in obtaining simple but realistic model approximations. The presence of bulky groups (as phenyl) can be ‘diluted’ by the presence of long alkyl chains in the same structure, whereas the lubricating effect of long alkyl chains can be hidden by very stiff backbones or by nanophase separations. The hybrid approach proposed can recognize these chemical features and quantify their relevance for estimating an alpha relaxation map area. It is important to highlight here that this knowledge is self-learned by the network, based only on the monomer chemical structure and the corresponding *T_g_* value, and that ECNLE theory converts this output into a relaxation map. This approach could substantially help gain both qualitative and quantitative insights into the behavior of polymeric materials, especially for properties that are difficult and/or expensive to measure.

## 4. Conclusions

The feasibility of joining artificial neural networks and theory into a hybrid system to provide an estimation of the temperature dependence of the polymer alpha relaxation, based only on the knowledge of the chemical structure of the monomer, has been demonstrated. The proposed method has been tested on a set of polyacrylates providing, for short side-chain polymers, an excellent agreement between the predicted and experimental temperature dependence of the relaxation times. This approach relies only on the knowledge of the monomeric chemical formula and does not require any kind of experimental measurements or calculations as input, and constitutes a valuable tool for boosting the scientific understanding of structure–property relationships.

## Figures and Tables

**Table 1:** ANN hyperparameters.

Item	Value
Data split ratio (train/test)	80/20
Dropout probability	0 to 0.3
Mini batch size	20
Learning rate	0.0001
Beta1 (Beta2)	0.99 (0.999)
# Hidden neurons (FC0–FC1)	30–20

## Data Availability

The data that support the findings of this study are available within the article and in the Supporting Information (SI) file.

## Supplementary Materials

The following are available online at https://www.mdpi.com/article/10.3390/polym14081573/s1, Table S1. The list of polyacrylates used in this work; Table S2. Parameters of the Vogel-Fulcher-Tammann (VFT) equation for the external control group; Figure S1. Schematic picture of the encoding process; Table S3. The number of filters and window sizes in the convolutional layers and the number of neurons in the fully connected layers; Figure S2. Shows the predicted vs experimental *T_g_* values for the internal subset of polyacrylates after finishing the training process; Table S4. ECNLE caltulations; Figure S3. Relative deviations (Experimental – Predicted) / Experimental (in %) histogram for the training and internal test sets (a). The chemical structures for those molecules with more significant relative deviations are shown in (b). Figure S4. Glass transition temperature dependence of the model adjustable parameter *a* for several polymers and glass formers.

## References

[1] Nakajima H., Dijkstra P., Loos K. (2017). The Recent Developments in Biobased Polymers toward General and Engineering Applications: Polymers That Are Upgraded from Biodegradable Polymers, Analogous to Petroleum-Derived Polymers, and Newly Developed. Polymers.

[2] Umoren S.A., Solomon M.M. (2019). Protective Polymeric Films for Industrial Substrates: A Critical Review on Past and Recent Applications with Conducting Polymers and Polymer Composites/Nanocomposites. Prog. Mater. Sci..

[3] de Leon A.C.C., da Silva Í.G.M., Pangilinan K.D., Chen Q., Caldona E.B., Advincula R.C. (2021). High Performance Polymers for Oil and Gas Applications. React. Funct. Polym..

[4] Wu X., Chen X., Zhang Q.M., Tan D.Q. (2022). Advanced Dielectric Polymers for Energy Storage. Energy Storage Mater..

[5] Wang Y., Ghanem B.S., Han Y., Pinnau I. (2022). State of the Art Polymers of Intrinsic Microporosity for High-Performance Gas Separation Membranes. Curr. Opin. Chem. Eng..

[6] Devaraju S., Alagar M. (2021). Polymer Matrix Composite Materials for Aerospace Applications. Encycl. Mater. Compos..

[7] Vidya, Mandal L., Verma B., Patel P.K. (2020). Review on Polymer Nanocomposite for Ballistic & Aerospace Applications. Mater. Today Proc..

[8] Gambino T., Alegría A., Arbe A., Colmenero J., Malicki N., Dronet S. (2020). Modeling the High Frequency Mechanical Relaxation of Simplified Industrial Polymer Mixtures Using Dielectric Relaxation Results. Polymer.

[9] Menard K.P., Menard N.R. (2015). Dynamic Mechanical Analysis in the Analysis of Polymers and Rubbers. Encycl. Polym. Sci. Technol..

[10] Capiel G., Miccio L.A., Montemartini P.E., Schwartz G.A. (2017). Water Diffusion and Hydrolysis Effect on the Structure and Dynamics of Epoxy-Anhydride Networks. Polym. Degrad. Stab..

[11] Otegui J., Miccio L.A., Arbe A., Schwartz G.A., Meyer M., Westermann S. (2015). Determination of Filler Structure in Silica-Filled SBR Compounds by Means of SAXS and AFM. Rubber Chem. Technol..

[12] Phan A.D., Schweizer K.S. (2018). Elastically Collective Nonlinear Langevin Equation Theory of Glass-Forming Liquids: Transient Localization, Thermodynamic Mapping, and Cooperativity. J. Phys. Chem. B.

[13] Phan A.D., Wakabayashi K. (2019). Effects of Cooling Rate on Structural Relaxation in Amorphous Drugs: Elastically Collective Nonlinear Langevin Equation Theory and Machine Learning Study. RSC Adv..

[14] Phan A.D., Knapik-Kowalczuk J., Paluch M., Hoang T.X., Wakabayashi K. (2019). Theoretical Model for the Structural Relaxation Time in Coamorphous Drugs. Mol. Pharm..

[15] Miccio L.A., Schwartz G.A. (2020). From Chemical Structure to Quantitative Polymer Properties Prediction through Convolutional Neural Networks. Polymer..

[16] Miccio L.A., Schwartz G.A. (2020). Localizing and Quantifying the Intra-Monomer Contributions to the Glass Transition Temperature Using Artificial Neural Networks. Polymer..

[17] Miccio L.A., Schwartz G.A. (2021). Mapping Chemical Structure-Glass Transition Temperature Relationship through Artificial Intelligence. Macromolecules.

[18] Weininger D. (1988). SMILES, a Chemical Language and Information System. 1. Introduction to Methodology and Encoding Rules. J. Chem. Inf. Comput. Sci..

[19] O’Boyle N.M. (2012). Towards a Universal SMILES Representation-A Standard Method to Generate Canonical SMILES Based on the InChI. J. Cheminform..

[20] Plazek D.J., Ngai K.L., Mark J.E. (2007). The Glass Temperature. Physical Properties of Polymers Handbook.

[21] Plastic Library, Chemical Retrieval on the Web, Crow. https://polymerdatabase.com.

[22] Bertinetto C., Duce C., Micheli A., Solaro R., Starita A., Tine M.R. (2007). Prediction of the Glass Transition Temperature of (Meth)Acrylic Polymers Containing Phenyl Groups by Recursive Neural Network. Polymer.

[23] Wypych G. (2016). Handbook of Polymers.

[24] Alkharusi H. (2012). Categorical Variables in Regression Analysis: A Comparison of Dummy and Effect Coding. Int. J. Educ..

[25] Srivastava N., Hinton G., Krizhevsky A., Sutskever I., Salakhutdinov R. (2014). Dropout: A Simple Way to Prevent Neural Networks from Overfitting. J. Mach. Learn. Res..

[26] Kingma D.P., Ba J. (2014). Adam: A Method for Stochastic Optimization. arXiv.

[27] He X., Wu J., Huang G., Wang X. (2010). Effect of Alkyl Side Chain Length on Relaxation Behaviors in Poly(n-Alkyl Acrylates) and Poly(n-Alkyl Methacrylates). J. Macromol. Sci. Part B Phys..

[28] Qin Q., McKenna G.B. (2006). Correlation between Dynamic Fragility and Glass Transition Temperature for Different Classes of Glass Forming Liquids. J. Non-Cryst. Solids.

[29] Menissez C., Sixou B., David L., Vigier G. (2005). Dielectric and Mechanical Relaxation Behavior in Poly(Butyl Methacrylate) Isomers. J. Non-Cryst. Solids.

[30] Sato A., Sasaki T. (2018). Cooperativity of Dynamics in Supercooled Polymeric Materials and Its Temperature Dependence Predicted from a Surface Controlled Model. Eur. Polym. J..

[31] Merino E.G., Atlas S., Raihane M., Belfkira A., Lahcini M., Hult A., Dionísio M., Correia N.T. (2011). Molecular Dynamics of Poly(ATRIF) Homopolymer and Poly(AN-Co-ATRIF) Copolymer Investigated by Dielectric Relaxation Spectroscopy. Eur. Polym. J..

[32] Xie S.J., Schweizer K.S. (2016). Nonuniversal Coupling of Cage Scale Hopping and Collective Elastic Distortion as the Origin of Dynamic Fragility Diversity in Glass-Forming Polymer Liquids. Macromolecules.

[33] Godard M., Saiter J. (1998). Fragility and Non-Linearity in Polymethyl (n-Alkyl) Acrylates. J. Non-Cryst. Solids.

[34] Calleja R.D., Jaime C., Sanchis M.J., Romcin J.S., Gargallo L. (1998). Dynamic Mechanical and Dielectric Relaxations in Poly(Pentachloropheny1 Methacrylate). Macromol. Chem. Phys..

[35] Calleja R.D., Jaime’s C., Sanchis-Sanchez M.J., Martinez-Piña F., Gargallo L., Radic D. (1997). Mechanical and Dielectric Properties of Bulky Side Chain Poly(Methacry1ates). Analysis of the Low Frequency Phenomena. 1: Poly(5-Lndanyl Methacrylate). Polym. Eng. Sci..

[36] Fredrickson G.H., Bates F.S. (1996). Dynamics of Block Copolymers: Theory and Experiment. Annu. Rev. Mater. Sci..

[37] Böhmer R., Ngai K.L., Angell C.A., Plazek D.J. (1993). Nonexponential Relaxations in Strong and Fragile Glass Formers. J. Chem. Phys..

[38] Diaz-Calleja R. (1991). Dielectric Relaxation Studies on Phenyl and Chlorophenyl Esters of Poly(Acry1ic Acid). Macromolecules.

[39] Floudas G., Štepánek P. (1998). Structure and Dynamics of Poly(n-Decyl Methacrylate) below and above the Glass Transition. Macromolecules.

[40] Garci A., Di R., Guzma J. (2000). Relaxation Behavior of Acrylate and Methacrylate Polymers Containing Dioxacyclopentane Rings in the Side Chains. J. Polym. Sci. Part B Polym. Phys..

[41] Sanchis M.J., Saiz E., Marti F. (2000). Dynamic Mechanical and Dielectric Relaxations of Poly (Difluorobenzyl Methacrylates). J. Polym. Sci. Part B Polym. Phys..

[42] García N., Compañ V., Díaz-Calleja R., Guzmán J., Riande E. (2000). Comparative Study of the Relaxation Behaviour of Acrylic Polymers with Flexible Cyclic Groups in Their Structure. Polymer.

[43] Balabin R.M. (2009). Enthalpy Difference between Conformations of Normal Alkanes: Raman Spectroscopy Study of n-Pentane and n-Butane. J. Phys. Chem. A.

[44] Wind M., Graf R., Renker S., Spiess H.W., Steffen W. (2004). Structure of Amorphous Poly-(Ethylmethacrylate): A Wide-Angle x-Ray Scattering Study. J. Chem. Phys..

[45] Wind M., Graf R., Renker S., Spiess H.W. (2005). Structural Reasons for Restricted Backbone Motion in Poly(n-Alkyl Methacrylates): Degree of Polymerization, Tacticity and Side-Chain Length. Macromol. Chem. Phys..

[46] Beiner M., Schröter K., Hempel E., Reissig S., Donth E. (1999). Multiple Glass Transition and Nanophase Separation in Poly(n-Alkyl Methacrylate) Homopolymers. Macromolecules.

[47] Beiner M., Kabisch O., Reichl S., Huth H. (2002). Structural and Dynamic Nanoheterogeneities in Higher Poly (Alkyl Methacrylate). J. Non-Cryst. Solids.

[48] Beiner M., Huth H. (2003). Nanophase Separation and Hindered Glass Transition in Side-Chain Polymers. Nat. Mater..

[49] Arbe A., Genix A., Arrese-Igor S., Colmenero J., Richter D. (2010). Dynamics in Poly (n-Alkyl Methacrylates): A Neutron Scattering, Calorimetric, and Dielectric Study. Macromolecules.

[50] Qazvini N.T., Mohammadi N. (2008). Segmental Dynamics in Net-Poly(Methyl Methacrylate)-Co-Poly(n-Butyl Acrylate) Copolymer Networks. J. Macromol. Sci. Part B Phys..

[51] Cangialosi D., Schwartz G.A., Alegría A., Colmenero J. (2005). Combining Configurational Entropy and Self-Concentration to Describe the Component Dynamics in Miscible Polymer Blends. J. Chem. Phys..

[52] Schwartz G.A., Alegría Á., Colmenero J. (2007). Adam-Gibbs Based Model to Describe the Single Component Dynamics in Miscible Polymer Blends under Hydrostatic Pressure. J. Chem. Phys..

