# Supplementary material for: Approaching Polymer Dynamics Combining Artificial Neural Networks and Elastically Collective Nonlinear Langevin Equation
Source: Polymers (Basel). 2022 Apr 12;14(8):1573. doi: 10.3390/polym14081573 (PMC9027377; doi:10.3390/polym14081573)
Supplement: Supplementary file 1 [file polymers-14-01573-s001.zip › polymers-1664857-supplementary.pdf]

polymers

MDPI

Supplementary Materials

Supplementary Materials: Approaching Polymer Dynamics Combining Artificial Neural Networks and Elastically Collective Nonlinear Langevin Equation

Luis Alejandro Miccio, Claudia Borredon, Ulises Casado, Anh D. Phan and Gustavo Ariel Schwartz

Section S1. Dataset

The list of polyacrylates used in this work, ordered by the value of the deviation in the predicted glass transition temperature, is shown in Table SI 1. The polymers in bold italics belong to the external control group.

Table S1. The list of polyacrylates used in this work.

Name	Experimental T_g [K]	Predicted T_g [K]	Relative Deviation [%]
Poly [11-(4-(4-dimethylaminophenyldiazenyl)phenoxy)undecyl methacrylate]	344	261.6	24.0
Poly [11-((4'-cyanobiphenyl-4-yl)oxy)-11-oxoundecyl methacrylate]	318	257.8	18.9
Poly [11-(4-(4-dimethylaminophenyldiazenyl)phenoxy)undecyl acrylate]	321	270.0	15.9
Poly [2-(2-(2-((4'-methoxybiphenyl-4-yl)oxy)ethoxy)ethoxy)ethyl acrylate]	344	294.0	14.5
Poly [2-((4'-methoxybiphenyl-4-yl)oxy)ethyl acrylate]	353	303.1	14.1
Poly [11-((4'-cyanobiphenyl-4-yl)oxy)undecyl acrylate]	303	260.8	13.9
Poly(2-cyanoheptyl acrylate)	389	339.1	12.8
Poly(terbutyl acrylate)	316	282.3	10.7
Poly [11-((4'-cyanobiphenyl-4-yl)oxy)undecyl methacrylate]	313	281.6	10.0
Poly(N,N-dibutylacrylamide)	333	306.4	8.0
Poly(2,2,2-trifluoroethyl acrylate)	***263***	***242.4***	***7.8***
Poly(ethyl methacrylate)	359	330.9	7.8
Poly(N-(1-methylbutyl)acrylamide)	380	359.0	5.5
Poly(2-hydroxyethyl methacrylate)	359	339.7	5.4
Poly(neopenthyl acrylate)	295	280.6	4.9
Poly(3,3-dimethyl-2-butylbutyl methacrylate)	381	362.5	4.9
Poly [2-(2-(2-(2-((4-cyanophenyldiazenyl)phenoxy)ethoxy)ethoxy)-ethoxy)ethyl methacrylate]	293	279.3	4.7
Poly [2-((4'-cyanobiphenyl-4-yl)oxy)ethyl acrylate]	323	308.3	4.6
Poly(biphenyl-4-yl methylacrylate)	413	394.5	4.5
Poly(4-cyano-3-thiabutyl acrylate)	249	239.3	3.9
Poly(octyl acrylate)	208	200.6	3.6
Poly(propyl acrylate)	236	228.0	3.4
Poly(terbutyl methacrylate)	391	379.0	3.1
Poly(hexyl acrylate)	216	209.5	3.0
Poly(methyl fluoromethacrylate)	357	346.4	3.0
Poly[ethyl 3-(acryloyloxy)benzoate]	297	288.8	2.8
Poly(4-methylphenyl methacrylate)	403	392.1	2.7
Poly(heptyl acrylate)	213	208.1	2.3
Poly(methyl chloroacrylate)	419	409.4	2.3

Poly(n-penthyl acrylate)	216	211.2	2.2
Poly(1H,1H-pentafluoropropyl acrylate)	247	242.2	1.9
Poly(3-ethoxypropyl acrylate)	218	213.8	1.9
Poly(butyl methacrylate)	***293***	***288.1***	***1.7***
Poly(2-methylphenyl methacrylate)	382	376.8	1.4
Poly(isopropyl methacrylate)	***359***	***354.1***	***1.4***
Poly[methyl 2-(acryloyloxy)benzoate]	319	315.0	1.3
Poly(1,3-dimethylbutyl acrylate)	258	255.3	1.0
Poly(N-terbutylacrylamide)	401	397.0	1.0
Poly(propyl methacrylate)	***308***	***305.1***	***1.0***
Poly(phenyl acrylate)	323	320.8	0.7
Poly(methyl methacrylate)	376	373.8	0.6
Poly(1H,1H,9H-hexadecafluorononyl methacrylate)	258	256.6	0.6
Poly(3-oxa-5-hydroxypentyl methacrylate)	278	277.0	0.4
Poly(2-ethoxyethyl acrylate)	223	222.2	0.3
Poly(2-methylpentyl acrylate)	235	234.3	0.3
Poly(N-butylacrylamide)	319	318.0	0.3
Poly [3-(4-(4-nitrophenyldiazenyl)phenoxy)propyl acrylate]	319	318.0	0.3
Poly(butyl chloroacrylate)	330	329.0	0.3
Poly(2-terbutylaminoethyl methacrylate)	306	305.1	0.3
Poly(3-thiabutyl acrylate)	213	212.4	0.3
Poly(2-chloroethyl methacrylate)	365	364.0	0.3
Poly(3-methoxybutyl acrylate)	217	216.5	0.2
Poly(2-cyanoethyl methacrylate)	364	363.2	0.2
Poly(2-heptyl acrylate)	235	234.5	0.2
Poly(2,3-dimethylphenyl methylacrylate)	398	397.1	0.2
Poly(2,3-dimethylphenyl 2-methylacrylate)	398	397.1	0.2
Poly[ethyl 4-(methacrylamido)benzoate]	441	440.1	0.2
Poly(4-methylphenyl acrylate)	316	315.3	0.2
Poly(4-chlorophenyl acrylate)	330	329.3	0.2
Poly [6-((4'-cyanobiphenyl-4-yl)oxy)hexyl acrylate]	315	314.4	0.2
Poly [5-((4'-cyanobiphenyl-4-yl)oxy)pentyl methacrylate]	333	332.4	0.2
Poly [4-(phenyldiazenyl)phenyl methacrylate]	374	373.3	0.2
Poly(methyl fluoroacrylate)	404	403.2	0.2
Poly[methyl 4-(methacrylamido)benzoate]	453	452.2	0.2
Poly(5-thiahexyl acrylate)	203	202.6	0.2
Poly [6-((4'-methoxybiphenyl-4-yl)oxy)hexyl acrylate]	338	337.4	0.2
Poly(benzyl acrylate)	279	278.5	0.2
Poly[methyl 3-(acryloyloxy)benzoate]	311	310.5	0.2
Poly [5-((4'-cyanobiphenyl-4-yl)oxy)pentyl acrylate]	313	312.5	0.2
Poly(2-phenylethyl acrylate)	270	269.5	0.2
Poly(propyl chloroacrylate)	344	343.4	0.2
Poly [methyl 3-(methacryloyloxy)benzoate]	345	344.5	0.2
Poly(8-cyano-7-thiaoctyl acrylate)	223	222.6	0.2
Poly [4-(4-cyanophenyldiazenyl)phenyl methacrylate]	363	362.4	0.2
Poly(6-cyano-4-thiahexyl acrylate)	215	214.7	0.2
Poly(4-methoxyphenyl acrylate)	324	323.5	0.2
Poly(cyanomethyl acrylate)	433	432.3	0.2
Poly(4-terbutylphenyl methacrylate)	371	370.5	0.1
Poly(1H,1H-undecafluorohexyl acrylate)	234	233.7	0.1
Poly [8-(4-(4-cyanophenyldiazenyl)phenoxy)octyl methacrylate]	308	307.6	0.1
Poly(3-dimethylaminophenyl acrylate)	320	319.5	0.1
Poly(5-cyano-3-thiapentyl acrylate)	214	213.7	0.1
Poly(ethyl ethacrylate)	300	299.6	0.1
Poly(2-cyanoisobutyl acrylate)	324	323.6	0.1
Poly(1H,1H-nonafluoropentyl acrylate)	236	235.7	0.1

Poly(3-chlorophenyl acrylate)	312	311.6	0.1
Poly(5-cyano-3-oxapentyl acrylate)	250	249.7	0.1
Poly(4-methoxyphenyl methacrylate)	379	378.5	0.1
Poly(3-oxa-butyl methacrylate)	289	288.6	0.1
Poly(4-thiahexyl acrylate)	197	196.8	0.1
Poly [2-(4-terbutylphenoxy)-2-oxoethyl methacrylate]	368	367.6	0.1
Poly(4-terbutylphenyl acrylate)	344	343.6	0.1
Poly [4-(4-(4-nitrophenyldiazenyl)phenoxy)butyl acrylate]	310	309.7	0.1
Poly(2-cyanoethyl acrylate)	277	276.7	0.1
Poly(1H,1H-undecafluoro-4-oxaheptyl acrylate)	205	204.8	0.1
Poly [4-(3-methoxy-3-oxopropyl)phenyl methacrylate]	341	340.7	0.1
Poly(2,4-dichlorophenyl methacrylate)	391	390.6	0.1
Poly(3,4-dimethylphenyl methylacrylate)	384	383.6	0.1
Poly(3,4-dimethylphenyl 2-methylacrylate)	384	383.6	0.1
Poly [6-((biphenyl-4-yl)oxy)hexyl acrylate]	321	320.7	0.1
Poly(5,5,5-trifluoro-3-oxapentyl acrylate)	235	234.8	0.1
Poly(4-cyanophenyl methacrylate)	428	427.6	0.1
Poly(fluoromethyl acrylate)	288	287.7	0.1
Poly(2-phenylethyl methacrylate)	299	298.7	0.1
Poly [2-(4-(phenyldiazenyl)phenoxy)ethyl methacrylate]	378	377.7	0.1
Poly(4-cyanophenyl acrylate)	363	362.7	0.1
Poly(4-cyanobenzyl acrylate)	317	316.7	0.1
Poly(3,5,5-trimethylhexyl methacrylate)	274	273.8	0.1
Poly(heptafluoro-2-propyl acrylate)	278	277.8	0.1
Poly(2-cyanoisopropyl acrylate)	339	338.7	0.1
Poly [6-(4-(4-methoxyphenyldiazenyl)phenoxy)hexyl acrylate]	319	318.8	0.1
Poly[methyl 4-(acryloyloxy)benzoate]	340	339.8	0.1
Poly(biphenyl-4-yl acrylate)	383	382.7	0.1
Poly [6-(4-(4-cyanophenyldiazenyl)phenoxy)hexyl acrylate]	300	299.8	0.1
Poly(3-thiapentyl acrylate)	202	201.9	0.1
Poly(1H,1H,5H-octafluoropentyl methacrylate)	309	308.8	0.1
Poly(biphenyl-2-yl acrylate)	378	377.8	0.1
Poly [6-(4-(4-nitrophenyldiazenyl)phenoxy)hexyl acrylate]	309	308.8	0.1
Poly(2-terbutylphenyl acrylate)	345	344.8	0.1
Poly(4-chlorophenyl methacrylate)	404	403.8	0.1
Poly[butyl 4-(methacrylamido)benzoate]	401	400.8	0.1
Poly(1H,1H,3H-hexafluorobutyl acrylate)	251	250.9	0.0
Poly(2,2,3,3,5,5,5-heptafluoro-4-oxapentyl acrylate)	218	217.9	0.0
Poly [2-(4-(4-pentiloxyphenyldiazenyl)-2-methylphenoxy)hexyl acrylate]	294	293.9	0.0
Poly(3,3-dimethylbutyl methacrylate)	318	317.9	0.0
Poly[butyl 4-(acryloyloxy)benzoate]	286	285.9	0.0
Poly [6-(4-(4-dimethylaminophenyl)diazenyl)phenoxy)hexyl acrylate]	327	326.9	0.0
Poly [4-(2-methoxy-2-oxoethyl)phenyl methacrylate]	354	353.9	0.0
Poly[4-(cyanomethyl)phenyl methacrylate]	401	400.9	0.0
Poly[methyl 4-(methacryloyloxy)benzoate]	379	378.9	0.0
Poly(1H,1H,5H-octafluoropentyl acrylate)	238	237.9	0.0
Poly(dodecyl methacrylate)	208	207.9	0.0
Poly(3-methylbutyl acrylate)	228	227.9	0.0
Poly(2,4-dichlorophenyl acrylate)	333	332.9	0.0
Poly(biphenyl-4-yl 2-methylacrylate)	413	412.9	0.0
Poly [4-(4-(4-cyanophenyldiazenyl)phenoxy)butyl methacrylate]	353	352.9	0.0
Poly(butyl cyanoacrylate)	358	358.0	0.0
Poly [4-(4-dimethylaminophenyldiazenyl)phenyl acrylate]	392	392.0	0.0
Poly(methyl atropate)	391	391.0	0.0

Poly[ethyl 4-(acryloyloxy)benzoate]	310	310.0	0.0
Poly [4-(4-dimethylaminophenyldiazenyl)phenyl methacrylate]	457	457.0	0.0
Poly(4,4,5,5-tetrafluoro-3-oxapentyl acrylate)	251	251.0	0.0
Poly(2-methylbutyl acrylate)	241	241.0	0.0
Poly(N-methyl-N-phenylacrylamide)	453	453.0	0.0
Poly(2-octyl acrylate)	228	228.0	0.0
Poly(1H,1H-heptafluorobutyl acrylate)	243	243.0	0.0
Poly(2-cyanobutyl acrylate)	384	384.1	0.0
Poly [6-(4-(4-dimethylaminophenyldiazenyl)phenoxy)hexyl methacrylate]	359	359.1	0.0
Poly [6-(4-(4-cyanophenyldiazenyl)phenoxy)hexyl methacrylate]	333	333.1	0.0
Poly(5,5,6,6,7,7,7-heptafluoro-3-oxaheptyl acrylate)	228	228.2	-0.1
Poly [6-(4-(4-methoxyphenyldiazenyl)phenoxy)hexyl methacrylate]	341	341.4	-0.1
Poly(acrylamide)	438	438.5	-0.1
Poly(N,N-diisopropylacrylamide)	393	393.5	-0.1
Poly [6-(4-(4-butoxyphenyldiazenyl)phenoxy)hexyl methacrylate]	352	352.4	-0.1
Poly(2,4-dimethylphenyl methylacrylate)	384	384.5	-0.1
Poly(butyl acrylate)	219	220.4	-0.7
Poly(3-methoxyphenyl methacrylate)	343	346.1	-0.9
Poly(4-benzoylphenyl methacrylate)	391	394.5	-0.9
Poly(3-methylphenyl acrylate)	298	301.4	-1.1
Poly(ethyl fluoromethacrylate)	316	319.7	-1.2
Poly(2-methylphenyl acrylate)	325	329.1	-1.2
Poly(2-cyanohexyl acrylate)	358	362.7	-1.3
Poly(isobutyl acrylate)	249	252.4	-1.3
Poly(pentyl methacrylate)	***270***	***274.3***	***-1.6***
Poly(N-isopropylacrylamide)	358	365.4	-2.1
Poly(2-ethylbutyl acrylate)	223	227.6	-2.1
Poly(2-ethylhexyl methacrylate)	263	268.5	-2.1
Poly(2-chlorophenyl methacrylate)	384	392.1	-2.1
Poly [2-((4'-cyanobiphenyl-4-yl)oxy)ethyl methacrylate]	368	376.4	-2.3
Poly(phenyl methacrylate)	***383***	***391.8***	***-2.3***
Poly[ethyl 2-(acryloyloxy)benzoate]	303	312.2	-3.0
Poly(octyl methacrylate)	253	261.5	-3.3
Poly(methyl acrylate)	281	290.7	-3.5
Poly(isobutyl methacrylate)	326	338.6	-3.9
Poly(dimethylaminoethyl methacrylate)	292	303.4	-3.9
Poly(4-cyanobutyl acrylate)	233	242.3	-4.0
Poly [2-(2-((4-cyanophenyldiazenyl)phenoxy)ethoxy)ethyl methacrylate]	314	327.5	-4.3
Poly(ethyl acrylate)	***249***	***262.1***	***-5.3***
Poly(2-ethylbutyl methacrylate)	284	299.3	-5.4
Poly [11-(4'-cyanobiphenyl-4-yl)undecyl methacrylate]	303	319.6	-5.5
Poly(hexyl methacrylate)	***268***	***283.2***	***-5.7***
Poly(3-methylphenyl methacrylate)	380	404.3	-6.4
Poly(2-chlorophenyl acrylate)	318	338.4	-6.4
Poly [methyl 2-(methacryloyloxy)benzoate]	337	358.7	-6.4
Poly(2-methoxyethyl acrylate)	223	237.6	-6.5
Poly(secbutyl methacrylate)	***333***	***355.6***	***-6.8***
Poly(3-methoxypropyl acrylate)	198	215.6	-8.9
Poly(benzyl methacrylate)	***327***	***361.9***	***-10.7***
Poly(N-octylacrylamide)	220	244.8	-11.3
Poly(neopentyl methacrylate)	312	347.7	-11.4

Poly(secbutyl chloroacrylate)	347	389.2	-12.2
Poly(N,N-dimethylacrylamide)	362	409.7	-13.2
Poly [2-(2-(2-((4-cyanophenyldiazenyl)phenoxy)ethoxy)ethoxy)ethyl methacrylate]	310	356.4	-15.0

Section S2. Experimental Dynamics

Table SI 2 shows, for the external control group, the parameters of the Vogel-Fulcher-Tammann (VFT) equation:

$$\tau(T) = \tau_0 exp\left(\frac{DT_0}{T - T_0}\right) \tag{1}$$

that fits the corresponding experimental dynamics together with the references the data were taken from. The following expression relates fragility (*m*) with parameter *D* in the VFT equation:

$$m = \left.\frac{\partial \log\left(\tau(T)\right)}{\partial(T_g/T)}\right|_{T=T_g} = \frac{DT_0T_g}{\left(T_g - T_0\right)^2}\log\left(e\right) \tag{2}$$

Table S2. Parameters of the Vogel-Fulcher-Tammann (VFT) equation for the external control group.

Name	T_g [K]	Fragility	D	T_0 [K]	τ_0 [s]	Ref
Poly (2,2,2 - trifluoroethyl acrylate)	269	109	3.90	237.8	1.2E-11	1
Poly (phenyl methacrylate)	383	56	17.06	266.7	1E-15	2
Poly (benzyl methacrylate)	327	94	8.64	267.9	1E-15	2
Poly (isopropyl methacrylate)	359	89	9.24	290.4	1E-15	2
Poly (secbutyl methacrylate)	333	130	5.89	289.5	1E-15	2
Poly (propyl methacrylate)	324	63	10.79	246.5	1E-13	3
Poly (butyl methacrylate)	304	67	9.96	235.9	1E-13	4
Poly (hexyl methacrylate)	281	45	17.27	187.3	1E-13	4
Poly (pentyl methacrylate)	291	49	15.24	201.9	1E-13	4
Poly (ethyl acrylate)	257	92	6.73	215.1	1E-13	4

Section S3. SMILES Codification

Figure SI 1 shows a schematic picture of the encoding process. Each molecular structure is converted into a SMILES string, which is transformed into a binary image by employing a dictionary of SMILES characters and one-hot encoding. The dimensions of the images are n_d columns (number of SMILES characters in the dictionary) and $npos_{max}$ rows (number of positions in the longest string of the dataset). In addition, mx stands for the number of polymers in the dataset.

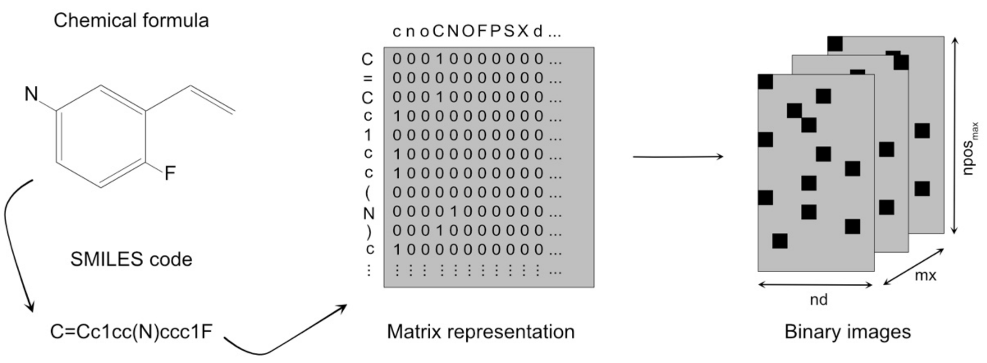

Figure S1. Schematic picture of the encoding process.

Section S4. ANN Architecture and Results

The neural network has been constructed using 6 convolutional and 2 fully connected layers, followed by an output linear regression layer. The number of filters and window sizes in the convolutional layers and the number of neurons in the fully connected layers are shown below.

Table S3. The number of filters and window sizes in the convolutional layers and the number of neurons in the fully connected layers.

Layer	Number of Filters/Neurons	Window Size
Convolutional 1	16	(1,1)
Convolutional 2	32	(2,2)
Convolutional 3	64	(2,2)
Convolutional 4	128	(2,2)
Convolutional 5	256	(2,2)
Convolutional 6	512	(2,2)
Flatten	-	-
FC_0	30	-
FC_1	20	-
Output layer	1	-

Figure SI 2 shows the predicted vs experimental T_g values for the internal subset of polyacrylates after finishing the training process. Grey dots represent the training set and red dots the validation set. The mean absolute percentage error for the training set is 4.3% and for validation is 8.5%.

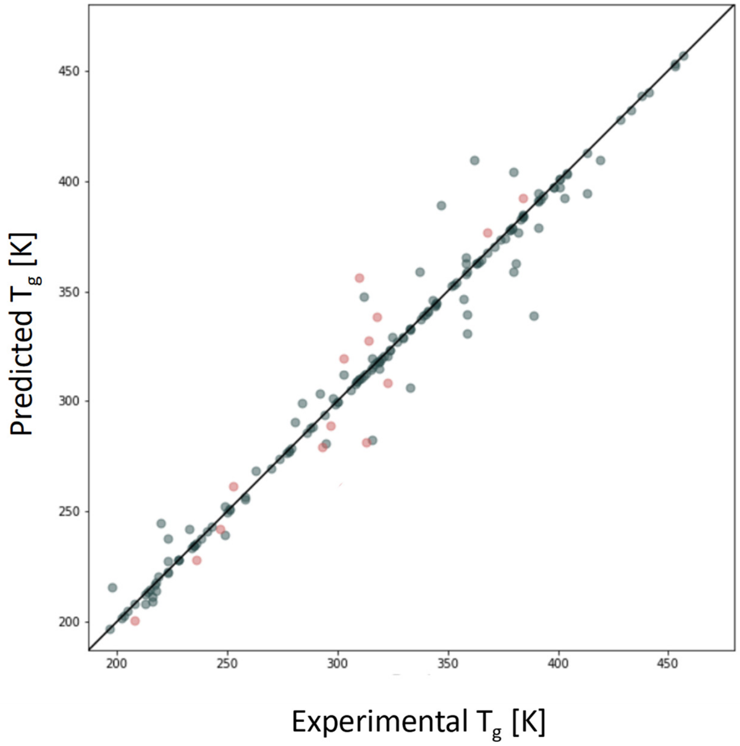

Figure S2. Shows the predicted vs experimental T_g values for the internal subset of polyacrylates after finishing the training process.

Section S5. Elastically Collective Nonlinear Langevin Equation (ECNLE)

As given in the main text, the temperature dependence of structural relaxation time is calculated using Kramer's theory

$$\frac{\tau}{\tau_s} = 1 + \frac{2\pi}{\sqrt{K_0 K_B}} \frac{k_B T}{d^2} exp\left(\frac{F_B + F_e}{k_B T}\right) \tag{3}$$

where $K_0, K_B, F_B/k_B T$ and $F_e/k_B T$ are determined from the dynamic free energy $F_{dyn}(r)$ and the radial distribution function, $g(r)$, and static structural factor, $S(q)$, of a hard-sphere fluid. The collective elastic energy contribution due to the long-range rearrangement of the molecules in the fluid, as a result of the escaping of a particle from its cage, is given by a sum over harmonic oscillator contributions whose shape is given by

$$F_e = 4\pi\rho \int_{r_{cage}}^{\infty} drr^2\, g(r) K_0 \frac{u^2(r)}{2} \tag{4}$$

where g(r) is the radial distribution function, K_0 can be interpreted as a spring constant at the localization length, and u(r) is a displacement field originating from the surface of the particle cage. In addition, we calculate the harmonic curvature at the barrier position K_B . We define the local quantities such as the localization length or the minimum position of $F_{dyn}(r)$, r_L , the barrier position or the local maximum position of $F_{dyn}(r)$, r_B , and the curvatures $K_0 = \left.\frac{\partial^2 F_{dyn}(r)}{\partial r^2}\right|_{r=r_L}$ and $K_B = \left|\left.\frac{\partial^2 F_{dyn}(r)}{\partial r^2}\right|_{r=r_B}\right|$. The short timescale τ_s is $g(d)^2 \tau_E$ with $g(d)$ being the contact number of the fluid. The functions of $g(r)$ and $S(q)$ can be determined by molecular dynamics (MD) simulations or Percus-Yevick theory. In our work, we employ the Percus-Yevick theory with only one input parameter: the volume fraction Φ.

All the above calculations give us the hard-sphere density dependence of alpha relaxation time. Quantitative comparisons between theoretical predictions and experiments require the thermal dependence of physical quantities. Thus, a thermal mapping constructed from the thermal expansion process is proposed, which is

$$T = T_g + (\Phi_g - \Phi)/\beta\Phi_0 \quad (5)$$

where β is the effective expansion coefficient, Φ_0 is the characteristic volume fraction, and Φ_g is the volume fraction at the glass transition defined by $\tau\left(\Phi_g\right) = 100\ s$. Values of all parameters needed for ECNLE calculations are expressed in Table SI 4.

Table S4. ECNLE caltulations.

Parameters.	Values
T_g	Given by experiments or simulation or ANN prediction in Kelvin
β	$12 \times 10^{-4}\ K^{-1}$
Φ_0	0.5
τ_E	$10^{-13}\ s$
Φ_g	0.6157

Section S6. Deviations and Chemical Structure

Figure S3 shows the relative deviations (Experimental – Predicted)/Experimental (in %) histogram for the training and internal test sets (a). The chemical structures for those molecules with more significant relative deviations are shown in (b).

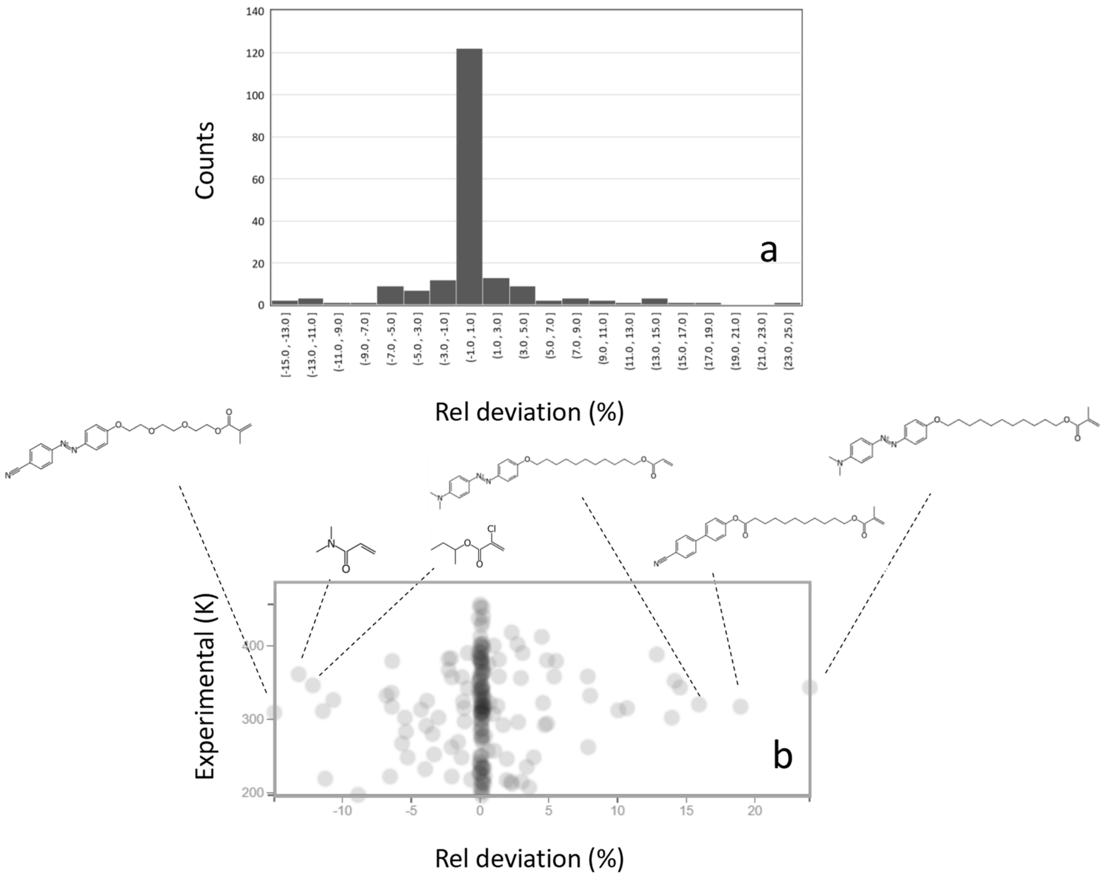

Figure S3. Relative deviations (Experimental – Predicted)/Experimental (in %) histogram for the training and internal test sets (**a**). The chemical structures for those molecules with more significant relative deviations are shown in (**b**).

Section S7. Parameter 'a' of ECNLE Model.

Figure S4 shows the glass transition temperature dependence of the model adjustable parameter *a* for several polymers and glass formers. The solid line represents the linear fit of the experimental data. Data were taken from reference 5.

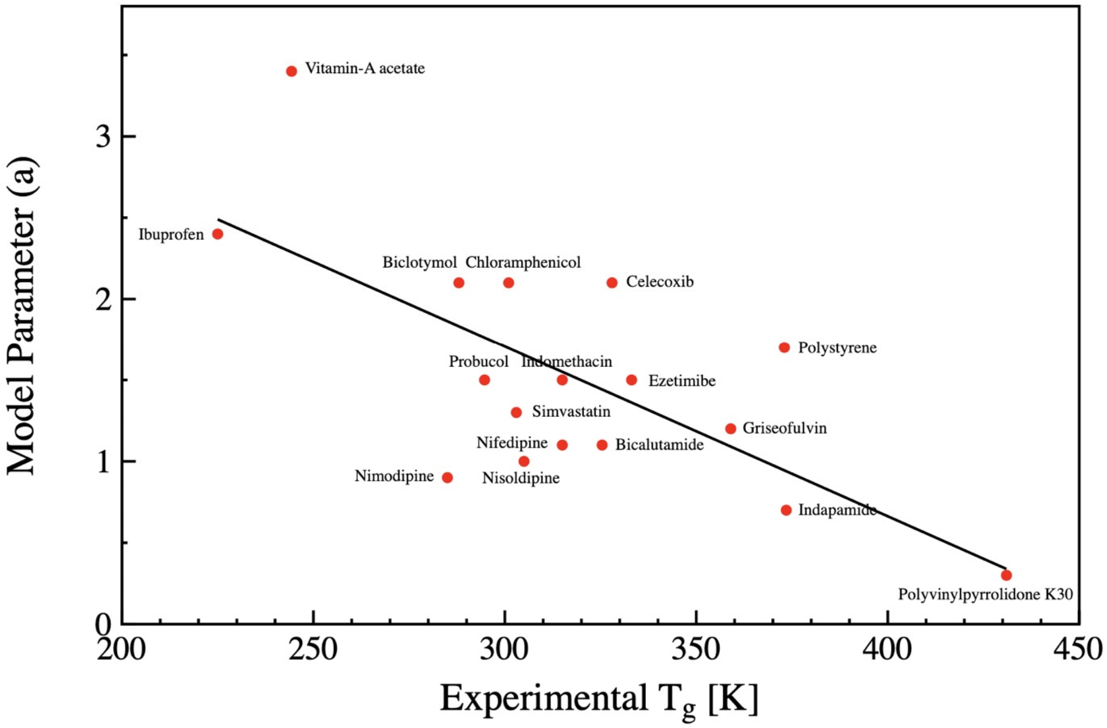

Figure S4. Glass transition temperature dependence of the model adjustable parameter *a* for several polymers and glass formers.
